# Association Between Acupuncture and Knee Surgery for Osteoarthritis: A Korean, Nationwide, Matched, Retrospective Cohort Study

**DOI:** 10.3389/fmed.2020.524628

**Published:** 2020-09-16

**Authors:** Byeong-Gu Gang, Joon-Shik Shin, Jinho Lee, Yoon Jae Lee, Hyun-Woo Cho, Me-riong Kim, Kyungwon Kang, Wonil Koh, Eun-Jung Kim, Yeoncheol Park, Dongwoo Nam, In-Hyuk Ha

**Affiliations:** ^1^Jaseng Hospital of Korean Medicine, Seoul, South Korea; ^2^Jaseng Spine and Joint Research Institute, Jaseng Medical Foundation, Seoul, South Korea; ^3^Haeundae Jaseng Hospital of Korean Medicine, Busan, South Korea; ^4^Department of Acupuncture & Moxibustion, College of Korean Medicine, Dongguk University, Gyeongju, South Korea; ^5^Department of Acupuncture & Moxibustion, Kyung Hee University Hospital at Gangdong, Seoul, South Korea; ^6^Department of Acupuncture & Moxibustion, College of Korean Medicine, Kyung Hee University, Seoul, South Korea

**Keywords:** knee osteoarthritis, knee surgery, acupuncture, National Health Insurance Service-National Sample Cohort (NHIS-NSC), respective cohort study

## Abstract

**Objectives:** The present study was undertaken to investigate the relationship between acupuncture therapy and surgery rate.

**Design:** Matched, retrospective cohort study.

**Materials and Methods:** From nationwide health insurance data (2002–2013 cohort data published by the National Health Insurance Service of Korea), patients with new cases of knee osteoarthritis that occurred between January 1, 2004 and December 31, 2010 were analyzed. Patients were divided into an acupuncture group (AG) and a control group (CG), based on records of acupuncture therapy. Propensity scores were calculated by using gender, age, income level, and Charlson comorbidity index (CCI), with the groups matched at a ratio of 1:3 (AG:CG). The final analysis period was 2 years after the first acupuncture therapy for AG and 2 years after initial diagnosis for CG; surgery rates were compared between the two groups. Stratified analyses were performed based on age, gender, and income level; sensitivity analyses were performed based on the frequency and duration of acupuncture therapy.

**Results:** Propensity score-matched AG and CG included 8,605 and 25,815 subjects, respectively. Post-matching surgery rates were 0.26 and 0.93% in AG and CG, respectively. For all age groups, AG showed a lower surgery rate than CG. In the analysis based on gender, the female group showed a significantly lower hazard ratio of 0.225. In analysis based on income level, the results of the entire group were significant, with the lower income group showing the lowest hazard ratio. In sensitivity analyses, AG tended to show a lower surgery rate than CG.

**Conclusions:** The present study demonstrated that acupuncture therapy is associated with a low rate of surgery for knee osteoarthritis. Additional studies are needed to support this conclusion.

## Introduction

Osteoarthritis is a degenerative disease associated with degeneration of the ligament and cartilage surrounding the joint; it exhibits gradual infiltration, loss of cartilage, structural change in the bone below the cartilage, and osteosclerosis, accompanied by bony spurs. Patients with knee osteoarthritis present with various symptoms, such as pain, muscle atrophy, stiffness, and limited range of motion (1). Pain is a major symptom associated with excessive joint use (2). According to the 2010 Global Burden Disease Study, the total social burden of hip and knee osteoarthritis was 11th in terms of years lived with disability and 38th in terms of disability-adjusted life years (3, 4).

Key treatment modalities recommended for patients with knee osteoarthritis in most international guidelines include patient education, exercise, and diet (5). When necessary, biomechanical methods involving a brace or insoles, or pharmacological approaches may be used, depending on the characteristics and preferences of individual patients (6, 7). Total knee replacement (TKR) is an effective treatment for patients with late-stage knee osteoarthritis (8), with an increasing number of TKR surgeries each year (9). However, even after surgery, ~20% of patients continue to experience persistent pain (10), in addition to persistent limitations in range of motion (11). Moreover, there have been reports of failed surgery involving sepsis, discontinuous extension, stiffness, femoral-tibial instability, patellar tracking, aseptic loosening, osteolysis, nearby fracture, and breakage of components (12).

Non-surgical treatments are typically administered to patients with early- and mid-stage knee osteoarthritis; among such methods, physical and acupuncture therapies are safe and effective (13). In particular, acupuncture therapy has a definitive effect on improving symptoms and movement (14, 15). In an overview of systematic reviews published in 2019, which examined the effects of acupuncture therapy on chronic knee pain due to osteoarthritis, acupuncture therapy was reported to demonstrate more total effective rate, short-term effective rate and less adverse reactions than western medicine of high quality outcomes (16).

Although the effects of acupuncture therapy on functional improvement and pain relief have been identified in cases of knee osteoarthritis, there have been no studies regarding the relationship between acupuncture therapy and knee surgery. Accordingly, the present study aimed to retrospectively review 2002–2013 Korean cohort data to investigate the relationship between acupuncture therapy and surgery rate for knee osteoarthritis.

## Materials and Methods

### Data Sources

The present study used 2002–2013 cohort data published in 2014 by the National Health Insurance Service of Korea. These data comprise random sampling of 1,025,340 people, representing ~2.2% of the total Korean population, for general research on the health status and health care utilization of all Koreans. The database included all medical claims filed from January 2002 to December 2010. In this study, diseases were classified according to the International Classification of Diseases, Ninth Revision, Clinical Modification (ICD-9-CM). For traditional Korean medicine (TKM), the Korean Standard Classification of Diseases (TKM), up to the end of 2009, was used. The present study was reviewed and approved by the Institutional Review Board of Jaseng Hospital of Korean Medicine (JASENG 2018-01-007).

### Study Population

**Figure 1:**
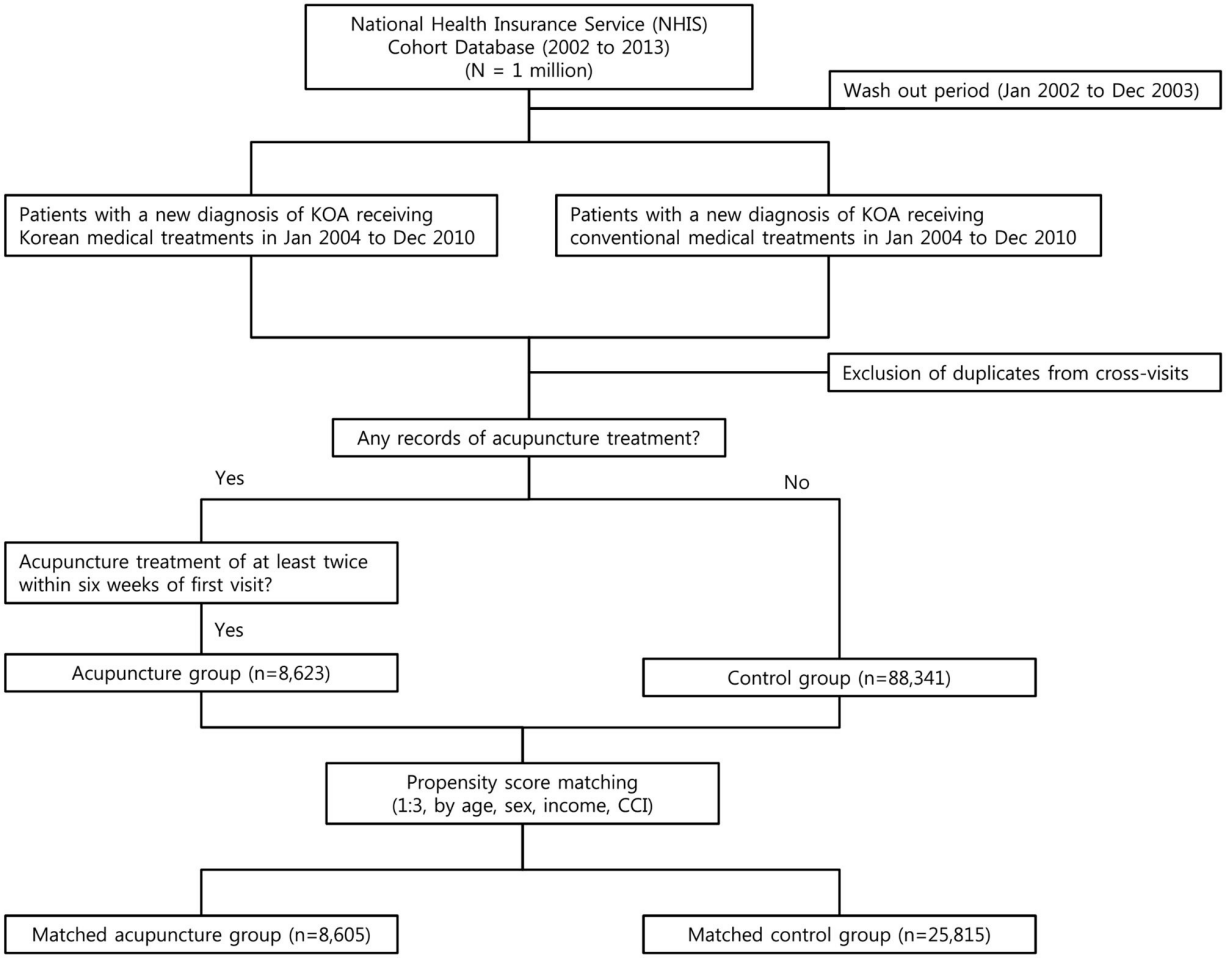
Flowchart of the study design. According to the propensity score matching (1:3, by age, sex, income, CCI), a total of 8,605 patients were included in the acupuncture group, and 25,815 patients were included in the control group.

Using the period from 2002 to 2003 as the wash-out period, the present study examined new cases of knee osteoarthritis that occurred between January 1, 2004 and December 31, 2010. M17^*^ was selected as the diagnosis representing knee osteoarthritis, based on review of existing studies and consensus among the researchers (17–19). For TKM, J18^*^, J19^*^, and H13.13 were used for cases up to December 31, 2009, in accordance with the revised TKM diagnoses; cases from January 1, 2010 and beyond used the standard medical diagnoses. Patients with the main or sub diagnosis mentioned above were considered new patients with knee osteoarthritis for each department; cases involving cross-treatment were excluded. Among patients who were identified according to these conditions, those with no record of acupuncture therapy after the initial diagnosis were selected as members of the control group (CG). Of the patients with records of acupuncture therapy, patients who were determined to show clinical significance, as agreed upon by the researchers, were considered candidates for the acupuncture group (AG). Subsequently, AG was established for patients who received at least 2 rounds of acupuncture therapy within 6 weeks after the initial diagnosis of knee osteoarthritis (Figure 1).

### Outcome Measures

Status of knee surgery was used as the factor for final analysis. For the definition of surgery, N0722^*^ (diagnostic condition), N2072^*^, N2077^*^, N2712^*^, N2717^*^, N0691^*^, N0692^*^, N0694^*^, N0702^*^ (diagnostic condition), N0707^*^ (diagnostic condition), E7500 (diagnostic condition), N0821^*^, N0822^*^, N0826^*^, and N0827^*^ were selected based on reviews of existing studies and consensus among the researchers (20, 21) The final analysis period was set to 2 years after the first acupuncture therapy for AG and 2 years after the initial diagnosis for CG. This was because the use of 2 years after the initial diagnosis for both groups may create a survival bias favorable to AG. Surgery rates that satisfied the aforementioned conditions were compared between the two groups.

### Data Analysis

To match the two groups, the present study used propensity score matching, generally used to eliminate confounding bias when comparing two non-randomized groups with different treatments. The present study calculated propensity scores based on gender, age, income level, and Charlson comorbidity index (CCI); considering the size of each group, the groups were matched at a ratio of 1:3 (AG:CG) (22). Data from subjects in each group who were not matched were discarded. Survival analysis was conducted for the surgery. Survival probability of acupuncture and control groups is presented as a Kaplan–Meier curve, and the difference between the two groups was tested using the log-rank test. Additionally, the number of patients at risk at each time point is presented. Cox proportional hazards regressions were used to estimate hazard ratios (HRs) and 95% confidence intervals (CI) for the relative risk of acquired incidence rate. The proportional hazards assumption was tested using Schoenfeld's global test. All analyses were performed using SAS statistical software (Version 9.3 for Windows; SAS Institute, Inc., Cary, NC, USA). Stratified analyses based on age, gender, and income level were performed to analyze the patterns by strata. Moreover, sensitivity analysis was performed to examine changes in results based on changes in the frequency and duration of acupuncture therapy specified in the study.

## Results

The numbers of unmatched subjects in AG and CG were 8,623 and 88,341, respectively. In the analysis by age, AG had the highest number of subjects (*n* = 3,035, 35.20%) in the 70–79 years' age group, while CG had the highest number of subjects (*n* = 23,616, 26.73%) in the 60–69 years' age group. With respect to gender, both groups had a higher percentage of females, with AG (*n* = 6,430, 74.57%) showing a higher percentage than CG (*n* = 53,188, 60.21%) (Table 1). The numbers of propensity score-matched subjects in the AG and CG groups were 8,605 and 25,815, respectively. For both AG and CG, the highest percentages of subjects belonged to the 70–79 years' age group, with 3,017 (35.06%) in AG and 9,000 (34.86%) in CG (Table 2). The median follow-up time of both groups was 730 days. The median duration of time to surgery was 144 days.

**Table 1:** Demographic characteristics of unmatched cohorts.

	**Acupuncture (*****n*** **=** **8,623)**		**Control (*****n*** **=** **88,341)**		*p*-value^*^
**Characteristics**	***n***	**(%)**	***n***	**(%)**	
**Age**					
20–29	290	3.36	3,851	4.36	<0.0001
30–39	627	7.27	7,793	8.82	
40–49	1,314	15.24	20,349	23.03	
50–59	1,031	11.96	14,360	16.26	
60–69	2,326	26.97	23,616	26.73	
70–79	3,035	35.20	18,372	20.80	
**Sex**					
Male	2,193	25.43	35,153	39.79	<0.0001
Female	6,430	74.57	53,188	60.21	
**Income**					
Lower	1,972	22.87	21,209	24.01	<0.0001
Middle	2,996	34.74	32,728	37.05	
Upper	3,655	42.39	34,404	38.94	
**CCI**					
0	3,070	35.60	37,129	42.03	<0.0001
1	2,641	30.63	28,324	32.06	
2	1,616	18.74	14,469	16.38	
3	785	9.10	5,602	6.34	
4	321	3.72	1,948	2.21	
5	129	1.50	641	0.73	
6	46	0.53	182	0.21	
7	13	0.15	38	0.04	
8	2	0.02	8	0.01	

* *p-values from the Chi-square test. CCI, Charlson Comorbidity Index*.

**Table 2:** Demographic characteristics of propensity score-matched cohorts.

	**Acupuncture (*****n*** **=** **8,605)**		**Control (*****n*** **=** **25,815)**		*p*-value^*^
**Characteristics**	***n***	**(%)**	***n***	**(%)**	
**Age**					
20-29	290	3.37	859	3.33	0.9989
30-39	627	7.29	1,888	7.31	
40-49	1,314	15.27	3,946	15.29	
50-59	1,031	11.98	3,091	11.97	
60-69	2,326	27.03	7,031	27.24	
70-79	3,017	35.06	9,000	34.86	
**Sex**					
Male	2,193	25.49	6,587	25.52	0.9545
Female	6,412	74.51	19,228	74.48	
**Income**					
Lower	1,970	22.89	5,877	22.77	0.7424
Middle	2,995	34.81	8,897	34.46	
Upper	3,640	42.30	11,041	42.77	
**CCI**					
0	3,070	35.68	9,320	36.10	0.8487
1	2,641	30.69	7,923	30.69	
2	1,616	18.78	4,859	18.82	
3	785	9.12	2,289	8.87	
4	321	3.73	981	3.80	
5	129	1.50	328	1.27	
6	35	0.41	90	0.35	
7	7	0.08	21	0.08	
8	1	0.01	4	0.02	

* *p-values from the Chi-square test. CCI, Charlson Comorbidity Index*.

In the overall analysis, AG and CG showed pre-matching surgery rates of 0.26 and 0.76% and post-matching surgery rates of 0.26 and 0.93%, respectively. AG and CG also showed HRs of 0.301 (0.197–0.461, 95% CI) and 0.273 (0.177–0.423, 95% CI), respectively (Table 3). The proportional hazards assumption was satisfied (Shoenfeld's global test, *p* = 0.307).

**Table 3:** Total and stratified analyses of the knee surgery rates in the acupuncture and control groups.

	**Acupuncture**			**Control**			**HR**	**95% CI**	p-value^*^
	***n***	**Cases**	**(%)**	***n***	**Cases**	**(%)**			
**TOTAL ANALYSIS**									
Unmatched	8,623	22	0.26	88,341	673	0.76	0.301	(0.197, 0.461)	<0.0001
Matched	8,605	22	0.26	25,815	240	0.93	0.273	(0.177, 0.423)	<0.0001
**STRATIFIED ANALYSIS**									
**Age**									
20–29	290	0	0.00	859	2	0.23	-	-	-
30–39	627	0	0.00	1,888	6	0.32	-	-	-
40–49	1,314	2	0.15	3,946	20	0.51	0.302	(0.071, 1.291)	0.1052
50–59	1,031	5	0.48	3,091	32	1.04	0.466	(0.182, 1.197)	0.1126
60–69	2,326	8	0.34	7,031	78	1.11	0.309	(0.149, 0.641)	0.0016
70–79	3,017	7	0.23	9,000	102	1.13	0.202	(0.094, 0.434)	<0.0001
**Sex**									
Male	2,193	7	0.32	6,587	42	0.64	0.500	(0.224, 1.112)	0.0892
Female	6,412	15	0.23	19,228	198	1.03	0.225	(0.133, 0.380)	<0.0001
**Income**									
Lower	1,970	4	0.20	5,877	59	1.00	0.201	(0.073, 0.555)	0.0019
Middle	2,995	11	0.37	8,897	77	0.87	0.420	(0.222, 0.790)	0.0071
Upper	3,640	7	0.19	11,041	104	0.94	0.203	(0.095, 0.437)	<0.0001
**CCI**									
0	3,070	6	0.20	9,320	75	0.80	0.243	(0.106, 0.557)	0.0008
1	2,641	10	0.38	7,923	63	0.80	0.475	(0.224, 0.925)	0.0286
2	1,616	3	0.19	4,859	47	0.97	0.191	(0.059, 0.614)	0.0054
3 and more	1,278	3	0.23	3,713	55	1.48	0.157	(0.049, 0.501)	0.0018

* *p-value from Cox-regression analysis adjusted by age, sex, income, and Charlson Comorbidity Index. CI, confidence interval; HR, hazard ratio; CCI, Charlson Comorbidity Index*.

**Figure 2:**
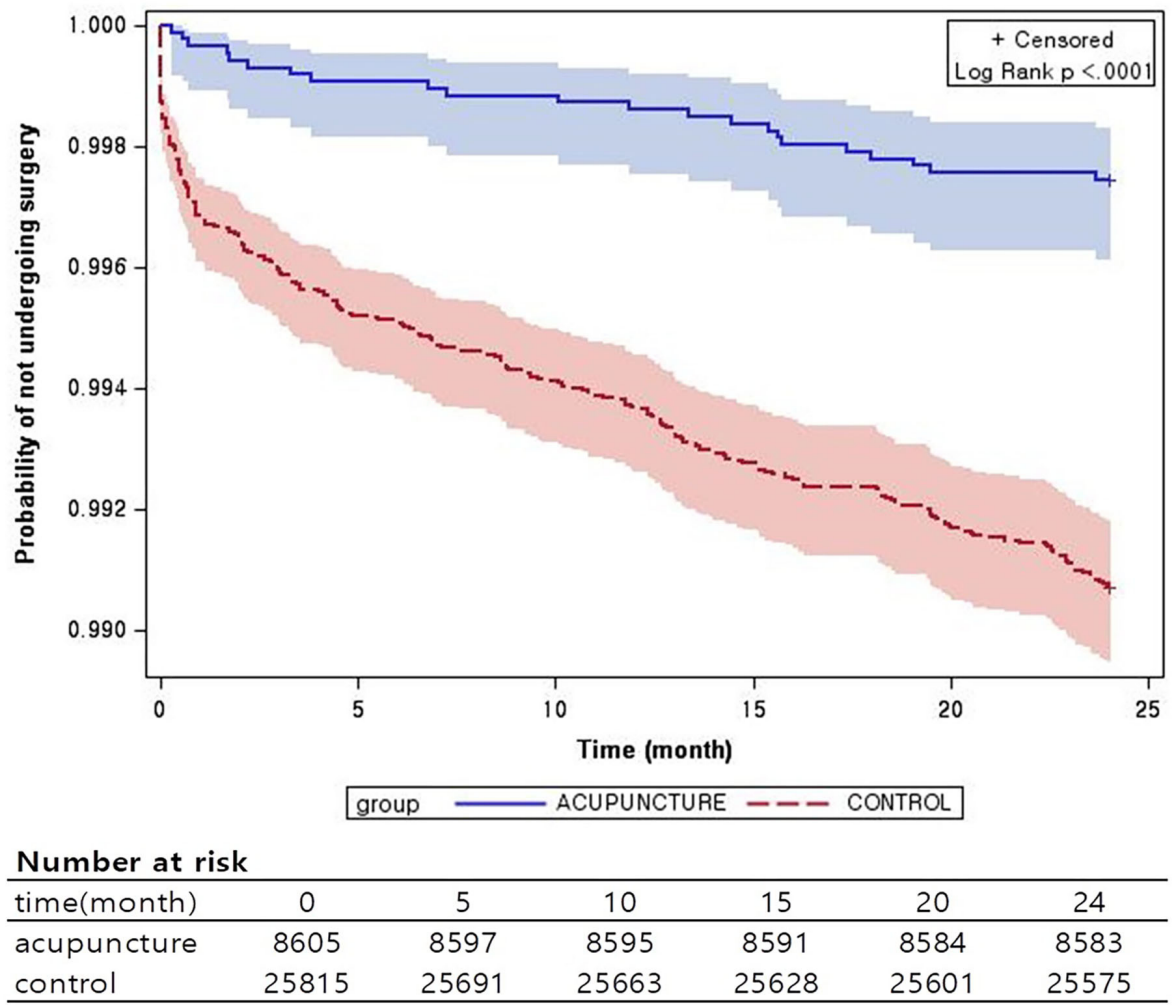
Kaplan–Meier survival estimates of knee surgery incidence. Probability of knee surgery with and without acupuncture treatment.

Stratified analyses were performed based on age, gender, and income level. In the analysis by age, the 70–79 years' age group showed the lowest HR of 0.202 (0.094–0.434, 95% CI). Among all ages, AG showed a lower surgery rate than CG, with an HR <1. However, the results were statistically significant only in the 60–79 years' age groups. In the 20–39 years' age groups, statistical comparisons could not be made because there were no cases that required surgery. In the 40–59 years' age group, HR was <1, but the results were not statistically significant. In the analysis by gender, males and females showed HRs of 0.5 (0.224–1.112, 95% CI) and 0.225 (0.133–0.380, 95% CI), respectively; the results were statistically significant only in females. In the analysis by income level, results for all groups were significant, but the lower income group showed the lowest HR, 0.201 (0.073–0.554, 95% CI) (Table 3, Figure 2).

The results of sensitivity analyses based on frequency and duration of acupuncture therapy are also presented. In all analyses, AG tended to show a lower surgery rate than CG, with an HR <1 (Table 4).

**Table 4:** Sensitivity analyses on knee surgery, according to the number of acupuncture sessions and the treatment period, in acupuncture and control groups.

	**Acupuncture**			**Control**			**HR**	**95% CI**	p-value^*^
	**n**	**Cases**	**(%)**	**n**	**Cases**	**(%)**			
**Number of sessions**									
2 and more	8,605	22	0.26	25,815	240	0.93	0.273	(0.177, 0.423)	<0.0001
3 and more	6,603	16	0.24	19,809	193	0.97	0.247	(0.149, 0.412)	<0.0001
4 and more	5,490	15	0.27	16,470	159	0.97	0.281	(0.166, 0.477)	<0.0001
5 and more	4,656	12	0.26	13,968	136	0.97	0.263	(0.146, 0.474)	<0.0001
**Treatment period (weeks)**									
1	8,315	20	0.24	24,945	237	0.95	0.251	(0.159, 0.397)	<0.0001
2	8,401	20	0.24	25,203	239	0.95	0.249	(0.158, 0.394)	<0.0001
3	8,470	20	0.24	25,410	239	0.94	0.249	(0.158, 0.394)	<0.0001
4	8,488	20	0.24	25,464	237	0.93	0.252	(0.159, 0.397)	<0.0001
5	8,536	22	0.26	25,608	238	0.93	0.276	(0.178, 0.427)	<0.0001

* *p-values from Cox-regression analysis adjusted by age, sex, income, Charlson Comorbidity Index. HR, hazard ratio; CI, confidence interval*.

## Discussion

The present study examined the association of acupuncture therapy with surgery rate in patients with knee osteoarthritis. In pre- and post-matching cohorts, AG showed a lower surgery rate than CG, with an HR <1. Analysis of surgery rates and HR values between the two groups showed that the results were significant in the age groups of 60–69 and 70–79 years, revealing appreciably lower HRs in older subjects. In the analysis by gender, females showed appreciably lower HRs. In sensitivity analyses based on frequency and duration of acupuncture therapy, the tendency of AG to have a lower surgery rate than CG was maintained, with HR <1.

In the pre-matching cohorts, primarily older age groups, females, and upper income level groups showed higher percentages; these patterns were maintained after matching. This may reflect differences in preferences based on age, gender, and income level with respect to TKM, including acupuncture therapy. In a study by Woo et al. that examined TKM preferences in the Korean population (23), acupuncture therapy was most well-known among users of TKM; most users of TKM, regardless of gender, age, education level, and TKM dependence, indicated that they had received acupuncture therapy, with preference for TKM especially high among the elderly. In addition, a 2006 study by Ock et al. (24) reported that preference for TKM was higher among females in Korea, with a large difference in utilization between males (69.3%) and females (80.3%). Such findings are supported by a 2008 study in Korea (25) and various foreign studies (26–28). In addition, a positive correlation was found between income level and use of CAM (29).

The number of subjects in the propensity score-matched groups tended to increase in age groups 50 years or older for both AG and CG, which may be because knee osteoarthritis is generally a degenerative joint disease (30). In both Tables 1, 2, the percentages of females were higher than those of males, which supported previous meta-analyses (31) which mentioned gender differences in the incidence rate of osteoarthritis, with a higher risk among females (32).

The major finding in the present study is that AG tended to show lower surgery rate than CG in the overall analysis and stratified analyses. However, because of no existing cases or small sample size (n), statistical significance could be found only in some groups.

The analysis results showed that pre- and post-matching surgery rates in CG increased from 0.76 to 0.93%, respectively, whereas HRs decreased from 0.301 to 0.273, respectively. This indicated that when the influences of other influencing factors of surgery rate were eliminated by propensity score matching, differences in surgery rate, in comparison with AG, were larger with statistically significant differences.

In the analysis based on age, the number of surgery cases among the 20–39 years aged AG group was zero. This may be because when AG patients were defined in the present study, disease codes corresponding to injuries were deemed unsuitable and excluded from patients with knee osteoarthritis. In AG, 50–59 years' age group showed the highest surgery rate, but the results were not statistically significant because the absolute number of patients was too small. The most noteworthy results were found in the 70–79 years' age group. In this age group, AG showed a relatively lower surgery rate than the other age groups (0.23%) and CG showed the highest surgery rate, while its HR was lowest (1.13%, 0.202, 0.094–0.434, 95% CI, *p* < 0.0001). This may be due to aforementioned preferences for TKM among the elderly. Moreover, older age groups tend to choose non-surgical treatment over surgery due to concerns regarding possible complications; thus, it is difficult to conclude that the findings in the present study solely reflected the effects of acupuncture therapy. A study by Belmar et al. (33) suggested that it may be more desirable to delay TKR in younger age groups, due to the possibility of re-operation, bone loosening, and pain due to over-use. Moreover, it might be better for older patients to delay or avoid surgery because there may be an increased risk of postoperative complications due to underlying diseases. Indeed, patients aged <50 years underwent surgery at a much lower rate than patients aged 60–69 years; patients aged ≥80 years underwent surgery at a lower rate than those aged 50–59 and 70–79 years.

In the analysis based on gender, AG showed a lower surgery rate than CG for both males and females. Surgery rates of females were statistically significant, with AG and CG showing 0.23% and 1.03%, respectively; the HR was 0.225 (0.133–0.380, 95% CI). This may be attributed to a greater number of female patients than male patients, as well as aforementioned gender differences in disease incidence. In the analysis based on income level, the results of the entire group were statistically significant.

To test the robustness of the analyses performed in the present study, sensitivity analyses based on frequency and duration of acupuncture therapy were performed. The results based on frequency and duration showed that surgery rate was higher in CG than in AG.

The noteworthy result in the analysis based on changes in frequency of acupuncture therapy was the difference between the ≥2 and ≥3 times groups. AG did not show a consistent tendency in surgery rate between the groups, whereas CG showed a noticeably higher surgery rate in the ≥3 times group than in the ≥2 times group; that rate was maintained as the frequency of acupuncture therapy increased. Moreover, the ≥3 times group showed the lowest HR, 0.247 (0.149–0.412, 95% CI). These results demonstrated that receiving acupuncture therapy ≥3 times within 6 weeks after the initial diagnosis was more closely associated with low surgery rates than receiving acupuncture therapy ≥2 times.

With respect to surgery rates based on duration of acupuncture therapy, AG showed a decrease in surgery rate from 0.26 to 0.24% when the duration decreased from 6 weeks to 1 week, while CG showed an increase from 0.93 to 0.95% for the same duration. Moreover, HRs showed a decreasing tendency. These results demonstrated that short-term intensive treatment after the initial diagnosis is associated with a low surgery rate.

The findings in the present study demonstrated that acupuncture therapy may be associated with a low surgery rate for knee osteoarthritis. As mentioned above, acupuncture therapy may show functional improvement and relieve pain in patients with knee osteoarthritis, which can reduce the need for surgery. Previous literature (34–37) mentioned that endogenous chemicals secreted during acupuncture therapy, such as enkephalin, dynorphin, and gamma aminobutyric acid, have a distinct effect on patients with chronic knee pain. However, the long-term pain control effect could not be verified; others have noted that the placebo effect may influence clinical outcomes (38). In other studies, compared with sham or no-treatment groups, acupuncture therapy did not show noticeable effects on functional recovery or pain relief in patients with chronic knee pain; thus, any clinical benefit may have been due to expectations by the patients. Indeed, patients who had positive expectations regarding acupuncture therapy showed better results than those who did not (39, 40). Consequently, the effect of acupuncture therapy on chronic knee pain remains controversial due to the limited number of studies and heterogeneity among studies (41–46). Moreover, currently suggested clinical guidelines have a contradictory view regarding the use of acupuncture therapy for knee osteoarthritis (6, 7, 47–49) Therefore, additional studies are needed to reaffirm the conclusions reached in the present study.

The present study used national cohort data, which had a large sample size that can represent the entire national population. Because the data were based on actual insurance claims, there is the advantage of no follow-up failure or loss, unlike other studies that require additional follow-up observations. While many studies have examined the association between acupuncture therapy and improvement in symptoms of knee osteoarthritis, there have been no studies regarding the correlation between acupuncture therapy and surgery rate for knee osteoarthritis. Moreover, the analyses in the present study were more significant due to additional sensitivity analyses regarding frequency and duration of acupuncture therapy.

The present study had some limitations. First, only surgery rate was used as the outcome for determining the association between acupuncture therapy and surgery. In doing so, the actual pre- and postoperative conditions of the patients could not be determined; thus, whether acupuncture therapy or surgery were helpful to patients could not be verified. Moreover, because new cases were defined relative to the date of initial diagnosis, there is a lack of information regarding whether the clinical conditions of the patients at the time required immediate surgery; whether the disease was in early stage; or what disease stage was present, based on radiologic findings. This suggests that the incidence of surgery may be influenced more by existing clinical conditions, compared with acupuncture therapy. Furthermore, the cohort data used in the present study did not consider items not covered under the Korean national health insurance system. Therefore, if the patient had received other treatments not covered by insurance, along with acupuncture therapy, the influence of such treatments could not be assessed. In addition, acupuncture therapy used in the present study was based on the curriculum used in traditional Korean medical colleges in Korea. Therefore, there may be differences with regard to acupuncture therapy used in other countries; differences may exist when applying these findings in other countries. Osteoarthritis is an age-related disease, but patients in their 20s and 30s were included in the analysis because the entire adult was subject to analysis. Although stratified analysis is presented by age, this limitation may weaken the meaning of the present results. Despite efforts to reduce selection bias through propensity matching, the entire health condition of the patient may not have been reflected because only the data in the claim data were checked. There may be concerns about the selection bias. Lastly, the number of patients in AG who underwent surgery was low; as a result, statistical significance could not be measured in some cases.

Knee replacement surgery is gradually becoming a more common treatment modality (8). This poses a significant economic burden in most health care systems, comprising $10.4 billion in the United States in 2008 (50). For this period, over 650,000 cases of TKR were performed in the US; between 2002 and 2005, a total of 103,601 cases of TKR were performed in Korea (51). However, after surgery, ~20% of the patients are known to suffer from persistent pain (52–54); some may require re-operation due to aseptic loosening, wear and tear of structures due to time and activities, and infection. Moreover, such adverse events may occur not only immediately after surgery, but at any time (55–58). Therefore, if an appropriate non-surgical treatment can be administered, postoperative pain and discomfort can be reduced, while reducing the economic burden associated with surgery within the overall health care system.

## Conclusions

The findings in the present study demonstrated that intervention by acupuncture therapy is associated with a low surgery rate in Korean patients with knee osteoarthritis. With the growing elderly population, the number of patients with knee osteoarthritis and the associated social burden are expected to increase. Therefore, strategies that can help alleviate the social and economic burdens of surgery, including TKR, are important for public health care policy makers; the findings in the present study may be helpful in that regard.

## Data Availability Statement

The present study analyzed the NHIS-NSC database, licensed by the Korean NHIS. Data are available from Korean NHIS upon submission of an appropriate study design.

## Ethics Statement

Due to the retrospective nature of this study, which utilized data with de-identified personal information, it was granted an exemption by the Institutional Review Board of Jaseng Hospital of Korean Medicine in Seoul, Korea (JASENG 2018-01-007). Informed consents process was waived by Institutional Review Board because the de-identified data was analyzed.

## Author Contributions

B-GG, YJL, M-rK, WK, and I-HH conceptualized and designed the study. J-SS, JL, KK, E-JK, YP, and DN acquired and analyzed the data. B-GG, YJL, KK, WK, and I-HH interpreted the results. B-GG, JL, YJL, M-rK, KK, and WK drafted the original article. J-SS, H-WC, E-JK, YP, DN, and I-HH critically revised the draft. All authors have read and approved of the final version.

## Conflict of Interest

The authors declare that the research was conducted in the absence of any commercial or financial relationships that could be construed as a potential conflict of interest.
